# Are Long-Lasting Insecticidal Nets Effective for Preventing Childhood Deaths among Non-Net Users? A Community-Based Cohort Study in Western Kenya

**DOI:** 10.1371/journal.pone.0049604

**Published:** 2012-11-19

**Authors:** Osuke Komazawa, Satoshi Kaneko, James K’Opiyo, Ibrahim Kiche, Sheru Wanyua, Masaaki Shimada, Mohamed Karama

**Affiliations:** 1 Department of Eco-Epidemiology, Institute of Tropical Medicine, Nagasaki University (NUITM), Nagasaki, Japan; 2 Graduate School of International Health Development, Nagasaki University, Nagasaki, Japan; 3 Centre for Public Health Research, Kenya Medical Research Institute (KEMRI), Nairobi, Kenya; 4 NUITM-KEMRI Project, Nairobi, Kenya; 5 Thomas Odhiambo Campus, Mbita, International Center of Insect Physiology and Ecology (ICIPE), Mbita, Kenya; Tulane University School of Public Health and Tropical Medicine, United States of America

## Abstract

**Background:**

Increasing the distribution and use of insecticide-treated nets (ITNs) in Sub-Saharan Africa has made controlling malaria with ITNs more practical. We evaluated community effects induced by ITNs, specifically long-lasting insecticidal nets (LLINs), under ordinary conditions in an endemic malaria area of Western Kenya.

**Methods:**

Using the database from Mbita Health and Demographic Surveillance System (HDSS), children younger than 5 years old were assessed over four survey periods. We analyzed the effect of bed net usage, LLIN density and population density of young people around a child on all-cause child mortality (ACCM) rates using Cox PH models.

**Results:**

During the study, 14,554 children were followed and 250 deaths were recorded. The adjusted hazard ratios (HRs) for LLIN usage compared with no net usage were not significant among the models: 1.08 (95%CI 0.76–1.52), 1.19 (95%CI 0.69–2.08) and 0.92 (95%CI 0.42–2.02) for LLIN users, untreated net users, and any net users, respectively. A significant increasing linear trend in risk across LLIN density quartiles (HR = 1.25; 95%CI 1.03–1.51) and a decreasing linear trend in risk across young population density quartiles among non-net user children (HR = 0.77; 95%CI 0.63–0.94) were observed.

**Conclusions:**

Although our data showed that current LLIN coverage level (about 35%) could induce a community effect to protect children sleeping without bed nets even in a malaria-endemic area, it appears that a better system is needed to monitor the current malaria situation globally in order to optimize malaria control programs with limited resources.

## Introduction

Malaria is the main cause of morbidity and mortality among children younger than 5 years old in Sub-Saharan Africa, and it is estimated to account for 16% (0·677 million) of all deaths in Africa [1]. According to the World Malaria Report 2011 [2], there were 216 million cases of malaria worldwide in 2010 and the number of deaths due to malaria was estimated to be around 655,000, with most victims in African regions being children younger than 5 years old. To avoid preventable deaths due to malaria among children, distribution of insecticide-treated nets (ITNs) has been increased in sub-Saharan Africa [3]. International funding for malaria control rose to US$ 1.8 billion in 2010, but World Health Organization estimates still fall short of the resources required for malaria control [4]. It is estimated that distribution of an adequate annual supply of ITNs to sub-Saharan Africa will cost more than US$ 6 billion [4], [5].

Randomized trials on the effectiveness of ITNs showed that ITNs provided 17% protective efficiency (PE) compared with the absence of nets and 23% PE compared with the presence of untreated nets [6]. In addition to these direct effects, ITNs also have community effects in the control of malaria. These community effects are a major advantage of ITNs because they reduce the risk of malaria parasite transfer from infected mosquitoes to humans and vice versa [7]. Several randomized controlled trials evaluated the community effects of ITNs, and all results demonstrated the effectiveness of ITNs in the regions surrounding the intervention areas, where bed net usage and high coverage were well controlled by community members [7]–[10]. However, these studies also showed a reduction in the protective effect of ITNs in areas that lacked bed net protection in terms of malaria morbidity and mortality as well as that the protective effect of ITNs ranged from several hundred meters to 1.5 kilometers from the area under ITN coverage [7]–[10].

The scaling up of ITNs distribution in sub-Saharan Africa has changed the focus of malaria control research, in which bed net usage and other factors are well controlled, to a more practical level, in which bed net distribution and usage are not well managed under routine situations [11]. Under these circumstances, continuous and systematic monitoring of the effect of ITN distribution is important to evaluate the effectiveness of scaling up ITN distribution worldwide [12]. However, only a few observational studies have been performed to evaluate the effectiveness of ITNs with respect to child mortality at a community level and under normal conditions [13], [14].

In this study, we sought to evaluate the effects of ITN, specifically long-lasting insecticidal nets (LLINs), and distribution by government and non-governmental organizations [15], focusing in particular on the effects on children sleeping without bed nets who are supposed to be protected by LLINs distributed around them. This study used data acquired by a Health and Demographic Surveillance System (HDSS) set up in Mbita, Western Kenya [16], [17], a highly endemic malaria area [18].

## Methods

### Study Site and Data Collection

The Mbita HDSS has been described in detail elsewhere [16], [17]. In brief, the study site of this HDSS is located on the shores of Lake Victoria in the Mbita district, Nyanza province, Kenya. This location is the highest endemic malaria area in Kenya [19]. It encompasses about 12,000 households and a population of 55,000. In 2010, the Mbita HDSS recorded 1351 live births and 416 deaths. Life expectancy was 61.0 years for females and 57.5 years for males. Under-5 mortality was 91.5 per 1,000 live births and Infant mortality was 47.0 per 1,000 live births. Most households (89%) used the lake as their main source of drinking water, and the proportion of households with toilets was lower than 40% [17]. Data were collected over a time interval of 5 to 6 weeks by 20 trained local data collectors using a Personal Digital Assistant (PDA) with a Global Positioning System (GPS) receiver to record geographic positions of households.

Between October 2008 and December 2010, four bed net usage surveys were conducted for all households in the Mbita HDSS area, during the routine follow-up rounds of the HDSS program. The first survey (period I) was conducted between October 14, 2008 and December 19, 2008; the second survey (period II) was between May 11, 2009 and June 4, 2009; the third survey (period III) was between January 7, 2010 and March 2, 2010; and the fourth survey (period IV) was between September 22, 2010 and December 3, 2010. The context of each survey was as follows. Field interviewers visually confirmed the type of bed net that was hanging inside each house. After checking the net tag that was stitched to the bed net, the field interviewer entered the bed net type into a PDA. Each bed net type was recorded as a LLIN, untreated net, or any bed net. For LLINs, the two brands that represent the only LLINs distributed and owned in the study area (Olyset® Net, Sumitomo Chemical Co.,Ltd., Japan; and Permanet ®, Vestergaard Frandsen, Switzerland), were recorded for periods II, III, and IV. During period I, however, these two brands of LLINs were just registered as an LLIN. Bed nets that the field interviewer could not confirm the bed net tag for bed net typing were recorded as any bed net. After bed net registration, the field interviewers asked who slept under each bed net on the night before the field interviewer’s survey date. These data were subsequently transferred from the PDA to a server and stored in an SQL database with other HDSS data sets. House IDs and resident IDs were assigned to both HDSS data and bed net survey data to link the data records from different HDSS datasets. Non-bed net users were not attached to any registered bed net in order to facilitate their identification from the dataset.

### Data Preparation

We retrieved data for children who were younger than 5 years old from the HDSS database. For each child, the person-years (PY) data were calculated from the bed net survey date for each of the four time periods until the earliest of the following four events occurred: 1) the next bed net survey date; 2) the out-migration date of the child to a new residence outside of Mbita; 3) the death date; or 4) the end of the study date (April 30, 2011). In terms of bed net use during the night, data were retrieved from the bed net survey data accumulated in the HDSS database. We assumed that the bed net use of each individual remained unchanged from one bed net survey to the following bed net survey.

**Figure 1:**
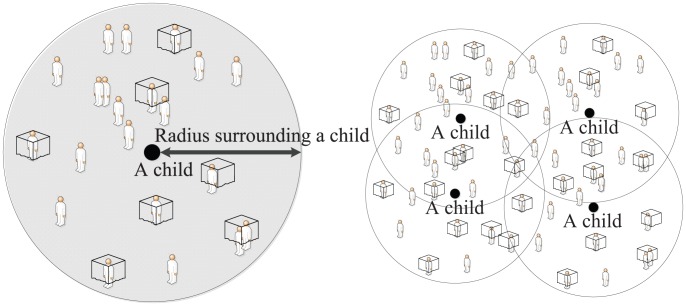
Schematic illustration for calculation of bed nets and population densities around a child. All individuals and all households within a given radius from 100 to 3,000 meters around each child were retrieved using the geographical position (longitude and latitude) of each household by GPS recorded in HDSS dataset in each survey period (period I, II, III, and IV).

After retrieval of data on each child from the dataset, we identified all individuals and all households within a given radius from 100 to 3,000 meters around each child using the command “globdist” [20] of STATA (version 12.1; Stata Corporation, TX, USA) to evaluate the effect of LLIN density around each child (Figure 1). The HDSS program stores the geographic position (longitude and latitude) of each household using GPS, so that this command (globdist) returns distance between the dwelling corresponding to each individual and the child. We counted bed net numbers used in all households surrounding the child using concentric circles within a radius of 100 meters to 3,000 meters, using 100 meter-intervals. These values represented the density of bed nets around the child. We also calculated the densities of bed nets in each survey period. Similarly, bed net densities around each child were calculated according to bed net type. For analysis, we classified bed net type into three groups: (1) LLIN, (2) untreated net, and (3) any bed net. Net usage proportions according to age and sex were calculated by dividing the number of children with a bed net by the total number of children in the time period. Moving averages of the adjacent five age groups were calculated to smooth out the values for the center age group [19], [21], [22]. To evaluate and control for the effect of the young population surrounding each child, we calculated the population between the age of 5 and 20 years for each child using the same procedure for bed net number calculation and added it to the analyses, because bed net usage is lower for this age group than for other age groups [23]. Furthermore, to remove the effect of townships where lower child mortality was expected due to easy access to health facilities, we created another data set (“remote dataset”) that excluded children whose house were located within 3 kilometers from the district hospital; or within 1 kilometer from health centers, dispensaries, and clinics. Because our study area was located in the rural area of Western Kenya, using this selection method allowed us to analyze only populations in remote areas. Corresponding to the remote dataset, the dataset of whole children including remote areas was named the “whole dataset”. Age and bed net use or type distributions are shown in Table 1. The radius range was decided by doubling the reported distance from a study on the community effects of ITNs using a randomized trial area; that study reported that the community effect of these nets was valid for up to 1.5 kilometers [7], and we studied the effects up to 3.0 kilometers, which is consistent with the reported flight range of malaria mosquitos [24].

**Table 1:** Age distributions (in months) of children by sex and bed net type.

1) Age (in months)								
	Whole dataset*					Remote dataset**		
	Period	Number of children	Mean	Std. Dev.		Number of children	Mean	Std. Dev.
Girls								
	I	5005	28.52	17.11		3040	28.40	17.21
	II	4766	30.12	16.98		2923	29.81	16.90
	III	4208	29.92	16.59		2681	29.53	16.52
	IV	4096	31.13	17.13		2622	31.26	17.11
Boys								
	I	5034	28.21	16.98		3028	28.37	17.04
	II	4818	29.79	16.78		2903	29.83	16.67
	III	4331	29.99	16.64		2657	30.24	16.46
	IV	4172	31.38	16.97		2603	31.63	17.18

2) Bed net type								
	Whole dataset					Remote dataset		
	Period	Number of children	Mean	Std. Dev.		Number of children	Mean	Std. Dev.
No user								
	I	7176	30.21	16.85		4589	30.25	16.94
	II	6847	32.37	16.51		4428	32.25	16.43
	III	4796	32.59	16.04		3212	32.76	15.92
	IV	4147	34.20	16.60		2969	34.81	16.60
LLIN user								
	I	1760	23.18	16.69		824	21.79	16.36
	II	1939	23.50	16.40		1021	21.49	15.57
	III	2527	25.54	16.77		1389	23.94	16.25
	IV	3233	27.84	16.93		1864	26.28	16.76
Untreated net user								
	I	1073	24.66	16.54		644	23.59	16.39
	II	350	26.63	16.38		180	25.51	15.95
	III	540	29.88	16.36		369	29.30	16.17
	IV	141	31.61	16.98		95	30.39	16.73
Any net user								
	I	30	24.57	17.84		11	24.09	17.10
	II	448	23.59	15.42		197	22.25	14.55
	III	676	27.81	16.54		368	27.78	16.34
	IV	747	29.68	17.11		297	30.50	16.89

* Whole dataset: the dataset of whole children.

** Remote dataset: Dataset that excluded children whose house were located within three kilometers from the district hospital; or within one kilometer from health centers, dispensaries, and clinics to remove the effect of townships where lower child mortality was expected due to easy access to health facilities.

### Basic Spatial Analysis

Distribution of death cases and LLIN distribution recorded in the HDSS database during the study periods were calculated with square grids of 500-meter sides generated by the “spgrid” command and smoothed by the kernel density estimation command “spkde” using STATA statistical software. Furthermore, spatial autocorrelations on death and LLIN distributions were assessed by computing Moran’s I using by the “spatgsa” command of the STATA program and the 500-meter square grids generated above.

### Statistical Analysis

To compare the risk of all-cause child mortality (ACCM) among children younger than 5 years old sleeping without a bed net with those using a LLIN, an untreated net, or any bed net, a Cox PH regression model was built controlling for age of children in months, socio-economic status of household, densities of LLINs, and population of young people within a certain radius (in meters) from a child in the HDSS area during the study period. The radii around each child were set from 100 meters to 3,000 meters with 100-meter intervals; as a result, we built 30 models to calculate the hazard risks for each of the 30 concentric circles. To select one best model from these 30 models, we applied the theory basis of the Akaike Information Criterion (AIC), which is defined as the best model that can be selected by two pieces of information, that is, the number of parameters in the statistical model and the maximized value of the likelihood function for the estimated model [25]. According to the AIC theory, the best model is that with the highest value of maximum likelihood among models, because we fixed the number of parameters for the models as follows: age in months as a time-varying covariate; principal component analysis (PCA)-based household asset index [26] for socio-economic status as a categorical variable after dividing into quartiles according to the distribution; densities of young people and bed net density around each child as a categorical variable after dividing into quartiles according to the distribution; and period as a categorical variable. The quartile values for LLIN and young population densities from the 100-meter radius model to the 3000-meter radius model are shown in Table S1. All categorized variables were transformed into dummy variables for analysis using the lowest categories as a reference group. The unit of time was months for the Cox PH models.

To treat age in months as a time varying covariate, a record of each child was split by month using the “stsplit” command of the STATA program. For example, a record of a child followed 2 years (24 months) was split into 24 records and age in months was assigned to each record. If the age in months was 2 months at entry point, the next record would be for 3 months of age, and the next record for 4 months of age. Because split records were identified by the child ID by the program, they were recognized as multiple record data for Cox PH analysis. Between two periods, split records will have the same values for other variables of the dataset.

Covariates of household asset index, densities of young people and bed net densities were divided into quartiles and hazard ratios (HRs) with 95% confidence intervals (CIs) were calculated using the lowest quartile as a reference. The PCA-based asset index scores used in the analysis are shown in Table S2. To assess the community effect by different LLIN densities around each child sleeping without a bed net, a second analysis was done following the same methods but using data only from children who slept without a bed net. To assess categorized exposure linear trends across the quartile categories of the young population and LLIN densities on child mortality, additional Cox PH models were made using the categorized variables of the young population and LLIN densities as a single independent variable [27]. For all data management and analysis, Stata version 12.1 (Stata Corporation, TX, USA) software was used.

### Ethical Considerations

Our HDSS program was approved by the Ethical Review Committee of Kenya Medical Research Institute (KEMRI SSC No. 1088) and the Ethical Committee of the Institute of Tropical Medicine, Nagasaki University (06060604). For participation in the HDSS program, the head of the household must give written informed consent for HDSS registration and follow-up on behalf of all household members including minors or children in the household. To maintain a good relationship between our HDSS program and communities, we have routine meetings for community sensitization, which improve consent rates for participation in the registration.

## Results

Details of the follow-up person-years, number of deaths by sex, and bed net type (for each period) are shown in Table 2. Between October 14, 2008, and April 30, 2011, 14,554 children were followed up and 250 deaths were recorded among them. The cohort was 19,908.0 person-years, and average length of follow-up was 1.4 years. The maximum follow-up period was 2.5 years and the minimum follow-up period was 1 day. The overall death rate was 12.6/1,000 person-years during all follow-up periods. The death rate was 12.2/1,000 person-years during period I, 10.8/1,000 person-years during period II, 15.6/1,000 person-years during period III, and 11.7/1,000 person-years during period IV. The crude mortality rate ratios for period II, III, and IV with respect to period I were 1.12 (95% CI: 0.77–1.63), 0.78 (95% CI: 0.55–1.11), and 1.03 (95% CI: 0.70–1.55), respectively. There were no significant differences between crude mortality rates in each period.

**Table 2:** Mortalities and person-years by bed net type and study period for the whole cohort.

Period	Sex	No net			LLINs			Untreated nets			Any bed net			Total		
		Person Years	Death cases	Death rates (per 1,000 PYs)	Person Years	Death cases	Death rates (per 1,000 PYs)	Person Years	Death cases	Death rates (per 1,000 PYs)	Person Years	Death cases	Death rates (per 1,000 PYs)	Person Years	Death cases	Death rates (per 1,000 PYs)
I	Girls	1699.5	15	8.8	425.9	6	14.1	258.9	3	11.6	7.1	0	0.0	2391.4	24	10.0
	Boys	1727.6	15	8.7	420.1	11	26.2	259.2	8	30.9	7.5	0	0.0	2414.4	34	14.1
	Both	3427.1	30	8.8	846.0	17	20.1	518.1	11	21.2	14.6	0	0.0	4805.8	58	12.1
II	Girls	1965.4	26	13.2	568.2	8	14.1	113.3	2	17.7	138.3	1	7.2	2785.1	37	13.3
	Boys	2019.0	17	8.4	607.6	6	9.9	98.8	0	0.0	127.8	1	7.8	2853.2	24	8.4
	Both	3984.5	43	10.8	1175.7	14	11.9	212.1	2	9.4	266.0	2	7.5	5638.3	61	10.8
III	Girls	1507.1	27	17.9	802.0	10	12.5	150.5	5	33.2	219.1	3	13.7	2678.7	45	16.8
	Boys	1498.5	24	16.0	848.2	13	15.3	203.9	0	0.0	210.1	2	9.5	2760.8	39	14.1
	Both	3005.6	51	17.0	1650.2	23	13.9	354.4	5	14.1	429.2	5	11.6	5439.5	84	15.4
IV	Girls	962.9	9	9.3	807.2	11	13.6	38.1	0	0.0	187.4	3	16.0	1995.6	23	11.8
	Boys	996.8	9	9.0	816.5	14	17.1	34.7	0	0.0	180.7	1	5.5	2028.7	24	11.8
	Both	1959.7	18	9.2	1623.7	25	15.4	72.8	0	0.0	368.1	4	10.9	4024.3	47	11.7
Total	Girls	6134.9	77	12.6	2603.3	35	13.4	560.8	10	17.8	551.9	7	12.7	9850.8	129	13.1
	Boys	6242.0	65	10.4	2692.4	44	16.3	596.7	8	13.4	526.1	4	7.6	10057.1	121	12.0
	Both	12376.9	142	11.5	5295.7	79	14.9	1157.4	18	15.6	1077.9	11	10.2	19908.0	250	12.6

**Figure 2:**
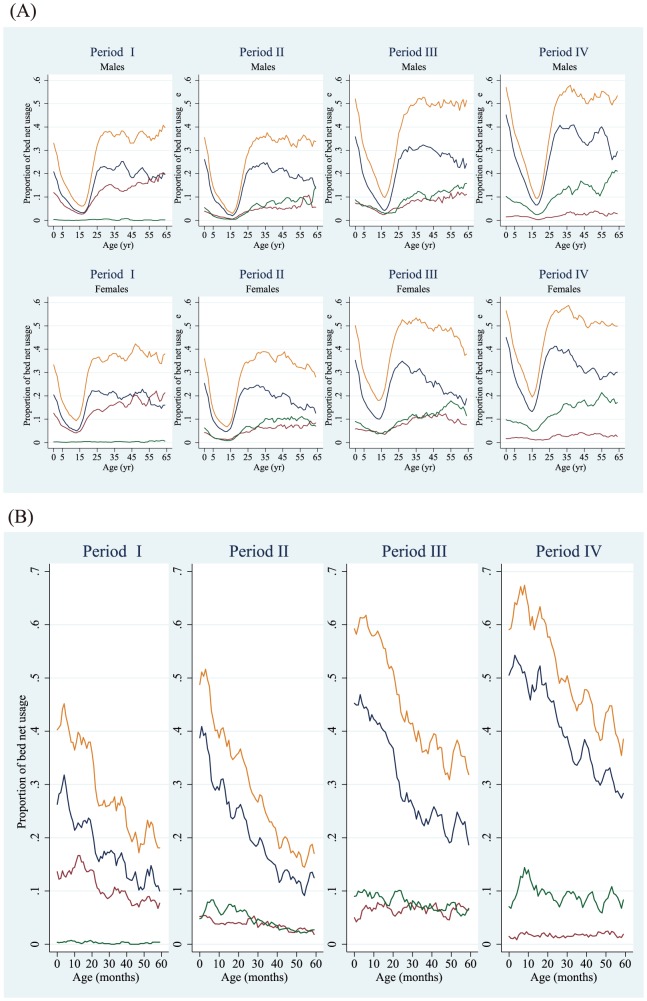
Bed net usage proportions during survey periods among individuals younger than 65 years (A) and among children younger than 5 years (B), according to the type of bed net used. Orange line, total bed nets; blue line, long lasting insecticide nets (LLIN); green line, untreated nets; dark red line, any bed net. Moving averages were calculated using five age groups to create graphs with smooth lines. Period I = October 14, 2008, to December 19, 2008; period II = May 11, 2009, to June 4, 2009; period III = January 7, 2010, to March 2, 2010; and period IV = September 22, 2010, to December 3, 2010.

The proportions of total net usage and LLIN usage improved over time among children younger than 5 (Figure 2-A). For girls younger than 5, the proportions of total net usage and LLIN usage increased by 33.3% and 20.4% in period I, by 35.9% and 25.4% in period II, by 50.1% and 35.3% in period III, and by 56.5% and 45.0% in period IV, respectively. For boys younger than 5, the proportions of total net usage and LLIN usage increased by 30.4% and 18.9% in period I, by 31.8% and 22.1% in period II, by 46.6% and 32.0% in period III, and by 53.7% and 42.6% in period IV, respectively. The tendencies observed for bed net usage proportions by age were almost the same for each time period and sex. Among females, the lowest bed net-use proportions and LLIN were recorded at 13 years of age: 9.3% and 5.0% in period I; 6.7% and 5.0% in period II; and 18.1% and 10.1% in period III, respectively. In Period IV, the lowest bed net-use proportions recorded were for 15-year-old females: total net use was 19.5% and LLIN use was 13.2%. For males, the ages recorded for lowest net-use proportions were older than those for females. In period I and II, the age associated with the lowest bed net-use proportions and LLIN proportions were 16 years: 6.1% and 3.3% in period I; and 3.0% and 2.0% in period II, respectively. In period III, the age associated with the lowest bed net-use proportion and LLIN proportion was 17 (9.8% and 4.3%, respectively) and in period IV, it was 18 (9.4% and 7.6%, respectively). Subsequently, the bed net-use proportions increased and they came to a plateau of 25-year-old males. Proportions of “any bed net” increased according to older age groups (Figure 2-A). This was due to lower confirmation rates for bed net tags in elder age groups compared with households with children. Because LLIN type bed nets were distributed in the area during the study periods, most bed nets registered as “any bed net” could be assumed to be LLINs. Under this assumption, the LLIN coverage rates for the entire population in the area were 34.5%, 26.0%, 41.2%, and 40.7% for periods I, II, III and IV, respectively (35.3% on average).

Bed net-use proportions for children younger than 5 years are shown in Figure 2-B. From 0 months to 59 months of age, the bed net-use proportions decreased in a linear fashion during all four periods. However, the bed net-usage proportion improved over time. For example, bed net-use proportions (total net use and LLIN use) for neonates improved by 40.3% and 26.3% during period I, 48.8% and 38.8% in period II, by 59.2% and 45.2% during period III, and by 59.1% and 50.5% during period IV, respectively. For children at 59 months of age, the bed net-usage proportions gradually improved by 18.1% and 10.0% during period I, by 17.0% and 12.5% during period II, by 31.8% and 18.7% during period III, and by 38.5% and 28.3% during period IV, respectively.

### Spatial Distribution of Death Cases and LLINs

**Figure 3:**
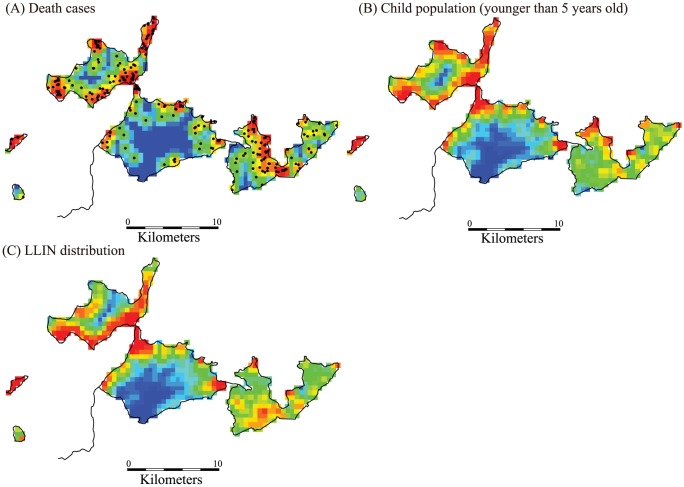
Spatial distribution of death cases (A), population of children younger than 5 years old (B), and long lasting insecticide net distribution (C) in the study area across the study period. Kernel estimation of the probability density function for each grid was calculated in terms of death cases, child population and long lasting insecticide treated nets using the quartic kernel function with the fixed bandwidth of 1000 meter. For visualizing the estimated probabilities by the kernel estimation, the probabilities were classified into 20 groups and describes on the grid of the map. The 20 groups were colored by rainbow color: the lowest group in blue, the middle group in green, and the highest group in red. Black dots in (A) are observed death cases.

The spatial distribution of death cases among the cohort during the study and LLIN distribution smoothed by kernel density estimation are shown in Figure 3. Because boundaries of villages in the study area were geographically unclear, square grids with 500 meter sides were used to show the distributions. Results of spatial autocorrelation are shown in Table 3 using the global Moran’s index for clusters. Both child deaths and LLIN distributions showed statistically significant tendencies for geographical clustering; however, the index for child deaths showed extremely small values compared with that of LLIN distributions. This means that child deaths had lower regional clustering compared with LLIN distributions.

**Table 3:** Global Moran’s index for death rates of children younger than 5 years old and long lasting insecticide net distribution across the 500-meter grids*.

Period	Moran’s index	*P*-value
1) Death rates		
I	0.002	0.122
II	0.008	0.003
III	0.002	0.141
IV	0.004	0.042
2) Long lasting insecticide nets		
I	0.077	0.000
II	0.081	0.000
III	0.073	0.000
IV	0.073	0.000

* Same 500-meter grids were used as Figure 2.

### Results from Cox PH Models

The adjusted HRs on ACCM among net user children compared with non-net user children were analyzed to evaluate the community effect induced by LLINs surrounding children using two datasets: a dataset including all children (“whole dataset”) and the dataset restricted only to children who lived far from health facilities (“remote dataset”). In the analysis using the whole dataset, the best fit model among the 30 models was the 700-meter radius model. HRs on ACCM were not significantly different between no net users and the other three net user groups; 0.92 (95%CI 0.69–1.24), 1.05 (95%CI 0.64–1.73), and 0.72 (95%CI 0.38–1.34) for LLIN users, untreated net users, and any net users, respectively. In analyses using the remote dataset, the best fit model was the 2500-meter radius model and the HRs on ACCM were 1.08 (95%CI 0.76–1.52), 1.19 (95%CI 0.69–2.08), and 0.92 (95%CI 0.42–2.02) for LLIN users, untreated net users, and any net users, respectively. The HRs of mortalities in different radius models from 100 meters to 3000 meters were relatively stable and were close to those of the best fit model for both datasets.

**Figure 4:**
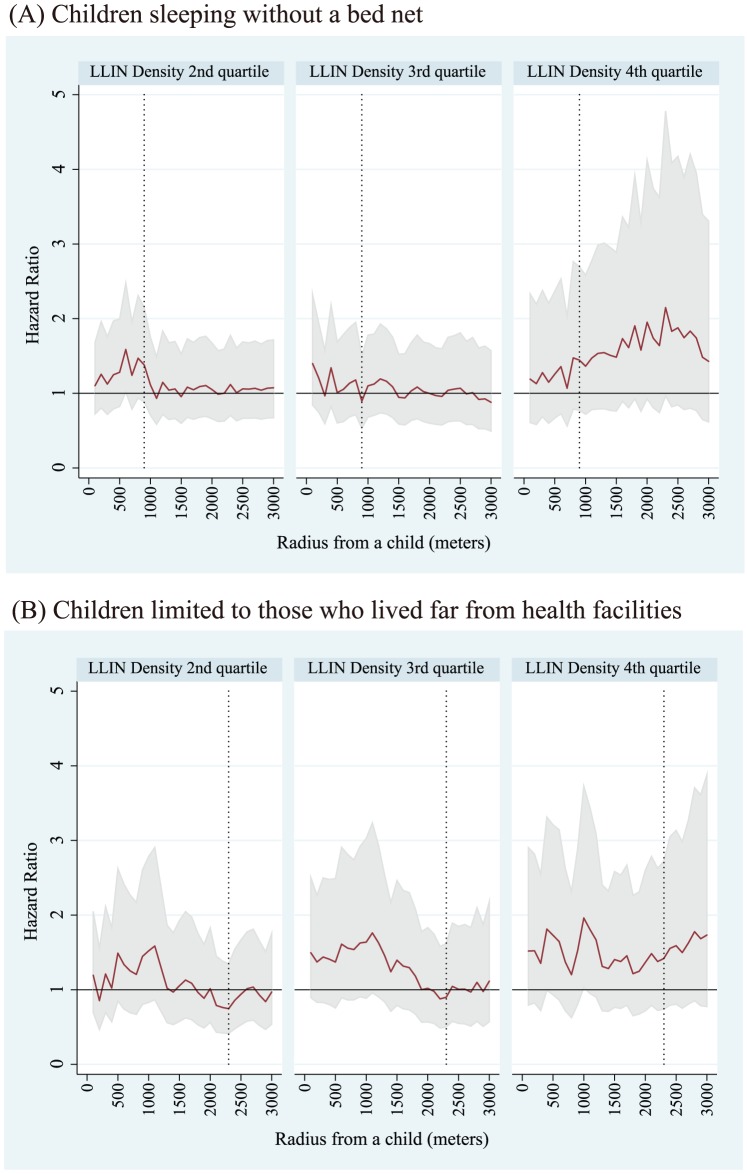
Adjusted hazard ratios (HRs) of densities of long lasting insecticide nets (LLINs) on all causes of child mortality (ACCM) for children sleeping without a bed net using the whole dataset ^*^(A) and for children limited to those who lived far from health facilities using the remote dataset^**^ (B). Reference is the lowest LLIN density quartile group of children. ^*^Whole dataset: Dataset covering the whole study area. ^**^Remote dataset: Dataset retrieved children of households located more than 3 kilometers from the district hospital or more than 1 kilometer from health centers and dispensaries in the area. Solid lines are point estimates of hazard ratios for Cox PH models within a radius from 100 meters to 3,000 meters and dotted vertical lines show the best fit model according to likelihood among models. Among the models in (A), the best fit mode was the 900-meter radius model (the dotted vertical line) and among the models in (B), it was the 2300-meter radius model (dotted vertical line). Gray bands indicate 95% confidence intervals for each point estimate.

**Figure 5:**
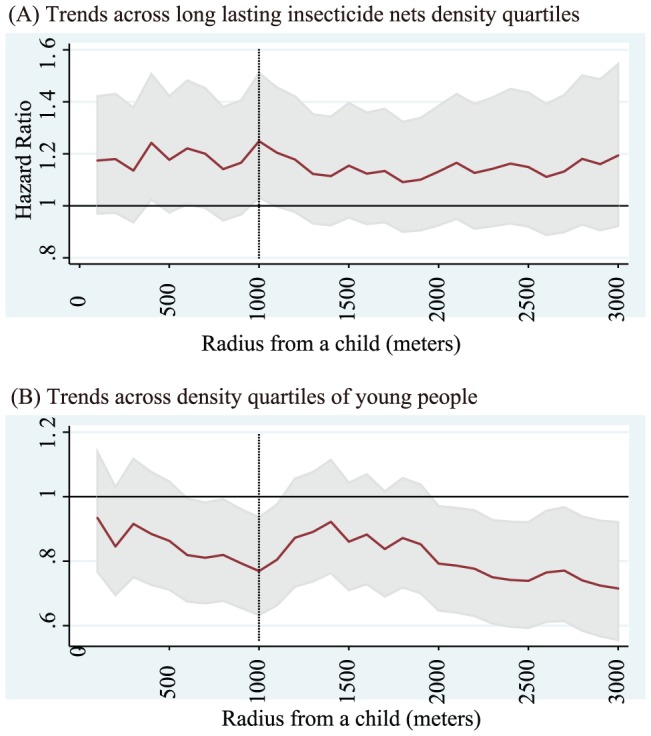
Trends in risk on all causes of child mortality (ACCM) for non-net user children across long lasting insecticide nets (LLINs) density quartiles (A) and those across density quartiles of young people (B) for children who live in a house far from health facilities (Remote dataset^*^). The best fit model was the 1000-meter radius model in trend analyses and the trend was 1.25 (95%CI 1.03–1.51) for LLIN density quartiles and 0.77 (95%CI 0.63–0.94) for density quartiles of young people. ^*^ Remote dataset: Dataset retrieved children of households located far from health facilities more than 3 kilometers from the district hospital or more than 1 kilometer from health centers and dispensaries in the area. Solid lines are point estimates of hazard ratios for Cox PH models within a radius from 100 meters to 3,000 meters and dotted vertical lines show the best fit model according to likelihood among models. Among the models both in (A) and (B), the best fit mode was the 1000-meter radius model (dotted vertical line). Gray bands indicate 95% confidence intervals for each point estimate.

Adjusted HRs on ACCM to evaluate the effect by LLIN densities surrounding a child who does not use bed nets for sleeping are shown in Figure 4. The best fit model was the 900-meter radius model for the whole dataset, and the 2300-meter radius model for the remote dataset. For the whole dataset, the HRs for children sleeping in the higher three quartiles of LLIN densities compared with the lowest quartile were 1.38 (95%CI 0.88–2.14) for the second quartile group, 0.89 (95%CI 0.53–1.50) for the third quartile group, and 1.44 (95%CI 0.77–2.70) for the fourth quartile group. For the remote dataset, they were 0.75 (95%CI 0.41–1.35) for the second quartile group, 0.90 (95%CI 0.50–1.62) for the third quartile group, and 1.42 (95%CI 0.74–2.72) for the fourth quartile group. The linear trend in risk for non-net user children across LLIN density quartiles using the remote dataset is shown in Figure 5 (A). The best fit model was the 1000-meter radius model and a significant trend for increasing risk was observed (HR = 1.25; 95%CI 1.03–1.51). The values of LLIN densities for each quartile for the 1000-meter radius model were 6.7 LLINs/km^2^, 14.0 LLINs/km^2^, and 23.6 LLINs/km^2^ for the 25, 50, and 75 percentiles, respectively.

**Figure 6:**
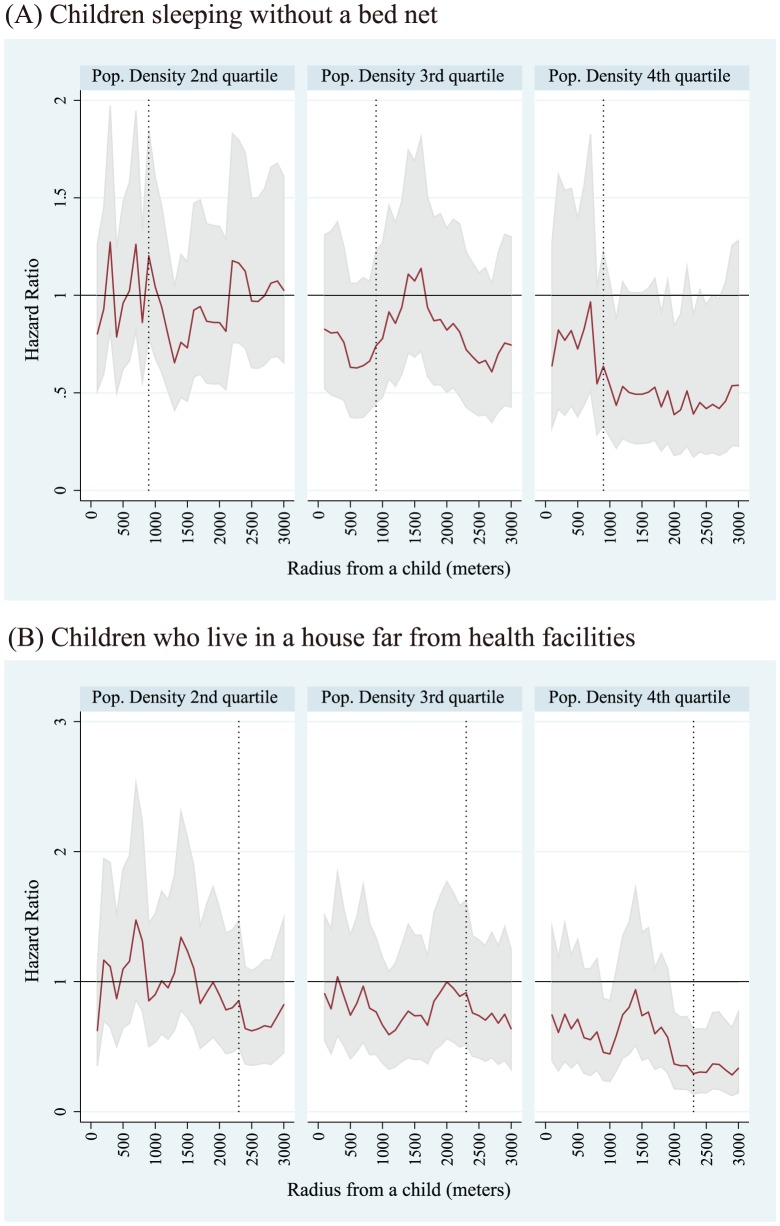
Adjusted hazard ratios (HRs) of densities of young population densities on all causes of child mortality (ACCM) for children sleeping without a bed net using the whole dataset ^*^ (A) for children limited to those who lived far from health facilities using the remote dataset^**^ (B). Reference is the lowest young population density quartile group of children. ^*^ Whole dataset: Dataset covering the whole study area. ^**^ Remote dataset: Dataset retrieved children of households located far from health facilities more than 3 kilometers from the district hospital or more than 1 kilometer from health centers and dispensaries in the area. Solid lines are point estimates of hazard ratios for Cox PH models within a radius from 100 meters to 3,000 meters and dot vertical lines show the best fit model according to likelihood among models. Among the models in (A), the best fit model was the 900-meter radius model (dotted vertical line) and among the models in (B), it was the 2300-meter radius model (dotted vertical line). Gray bands indicate 95% confidence intervals for each point estimate.

Adjusted HRs on ACCM to evaluate and control the effect by densities of young people surrounding children who do not use bed nets for sleeping are shown in Figure 6. The best fit model was the 900-meter radius model for the whole dataset, and the 2300-meter radius model for the remote dataset. For the whole dataset, the HRs for children sleeping in the higher three quartiles of densities of young people compared with the lowest quartile were 1.20 (95%CI 0.78–1.85) for the second quartile group, 0.74 (95%CI 0.45–1.23) for the third quartile group, and 0.64 (95%CI 0.33–1.22) for the fourth quartile group. For the remote dataset, values were 0.85 (95%CI 0.50–1.47) for the second quartile group, 0.91 (95%CI 0.51–1.63) for the third quartile group, and 0.29 (95%CI 0.14–0.63) for the fourth quartile group. The linear trend in risk for non-net user children across young population density quartiles using the remote dataset is shown in Figure 5(B). The best fit model was the 1000-meter radius model, and a significant trend for decreasing risk was observed (HR = 0.77; 95%CI 0.63–0.94).

When we assume “any bed nets” were LLINs, the results of Cox PH models using the remote data set after categorizing bed net type for each individual were not very different: 0.89 (95%CI: 0.67–1.18) for non-bed net user children compared with LLIN net user children. The HR for the trend in risk across LLIN density quartiles was 1.25 (95%CI: 1.032–1.51), and the HR for the trend in risk across young population density quartiles was 0.77 (95%CI: 0.63–0.94).

Household asset index was not statistically significant for any model, although it was kept as a controlling socioeconomic status factor in all analyses.

## Discussion

This observational study is based on the use of data acquired by a large group of HDSS data collectors to evaluate the effects of LLIN distribution on ACCM rates. This study is also based on normal conditions in a rural area of Africa, where net distribution and the instructions regarding bed net use are not systematic. In this sense, this study can be regarded as a good “surveillance program” to monitor ongoing malaria control efforts in Africa in ordinary conditions [12]. Our results can be summarized as follows: 1) during the study period, we observed no statistically significant increase or decrease in ACCM rates between LLIN users and non-net users for children younger than 5; 2) a significant increased linear trend in risk across LLIN density quartiles among non-net user children was observed (Figure 5), though pairwise comparisons to a reference group (lowest categorized group) were not significant (Figure 4); 3) a significant decreased linear trend in risk across quartiles of density of young people among non-net user children was observed (Figure 5), although only pairwise comparisons of children who live in the highest density of young populations to those who live in the lowest density space were significant (Figure 6). Considering that malaria is still endemic in the study area [18], [28], [29], the finding that there were no differences in ACCM rates between bed net users and non-bed net users (for children younger than 5) can be interpreted that LLINs are effective in reducing ACCM rates both for net users and non-net users by means of a community effect induced by ITN or LLIN distribution within communities.

Taking into consideration the estimated LLIN coverage of our study, a 35.3% on average LLIN coverage level could induce enough of a community effect to negate the need for further LLIN distribution. A mathematical model reported that slightly higher bed net usage rates were required to achieve communal effects to protect children who do not use bed nets with 35% and 65% coverage [30]. Furthermore, it also stated that emerging ITN technologies with long-lasting insecticidal properties would provide community effects at low coverage levels. Considering the limited resources available for malaria control, we should know the optimized coverage levels for ITNs or LLINs to prevent deaths due to malaria.

Although the community effect of LLINs affects the community as a whole, the effect might be different according to the density of LLINs in the surrounding areas. We hypothesized that the community effect of LLINs on children without bed nets would be more apparent if a child is sleeping with a higher density of surrounding LLINs, because we can expect fewer mosquitos around a child with a higher density of LLINs. Furthermore, we hypothesized that a child who sleeps with higher density of surrounding young people would have an increased risk of malaria infection, which leads to increased mortality, because young individuals often do not use bed nets at night, can be reservoirs of malaria parasites, and distribute the parasites to other surrounding children via mosquitos [19], [21], [22]. However the results of our analyses showed opposite results from the above hypotheses, that is, an increasing linear trend in risk of ACCM for higher LLIN densities and reduced linear trend in risk for higher densities of young people.

The increased risk of death for children sleeping without a bed net in a higher LLIN density circumstance might be explained by the escape behavior of mosquitos from houses with LLINs. Escaping mosquitos from houses with LLINs may increase the concentration of mosquitos in places where children are sleeping without bed net, even though they are in the vicinity of high LLIN densities. As a result, the risk of being bitten by a mosquito may increase. Some studies have reported that malaria mosquitos escape to outdoor locations and then bite humans who are sleeping outdoors, even in the vicinity of ITN usage [31]–[33].

A decreased risk of death among children without a bed net in a higher density young population also could be explained by the concepts in the theory of zooprophylaxis, that is, that mosquitoes are feeding on older children at a lower risk of mortality, which thereby grants some protection to younger children at a higher risk of mortality [34]–[36]. This effect remained even when we restricted our analysis to children who live far from a hospital and health facilities. In the study area, there are many fishery villages with high population densities. High population density areas are not always townships and communities with high socioeconomic status. A reduced effect of malaria transmission was reported in high human population density areas [37]. Young individuals who do not use bed nets can reduce the probability that other children without bed nets during the night will be bitten by mosquitos. As a result, the risk of malaria transmission and death might be reduced.

In this study, we applied concentric circles to calculate the number of LLINs and young populations surrounding a child, because this area must be defined to calculate the numbers and densities of LLIN and young populations. In other words, the concentric circles were used to define the exposure status for each child, which was used as an independent variable in the analytic models. In most studies that use concentric circles to examine health impacts, these circles are used to detect cases around the exposure point to calculate incidence or mortality, which is used as a dependent variable in analysis [38]; few studies use concentric circles to define exposure levels [39]. To define exposure level using concentric circles, the area or radius around the subjects becomes critical. We can set the radius around the subjects from zero to infinite; however, if we choose a large enough radius to cover the whole study area, the exposure level for all subjects becomes the same because each circle around each subject contains every exposure point in the area. Therefore, we made models using range of radii, from 100 meters to 3000 meters, and defined the best fit model using AIC. However, there still remains room for argument that the radius obtained in our study can be generalized for evaluating quantitatively the effect of exposure levels of LLIN and young population densities.

This study has some limitations. We assumed that the bed net status of each child remained unchanged from one survey to the next; however, this may not always be the case. Some children might receive a LLIN or bed net after a survey is completed, and some children might exit their bed net at night on one or more occasions before the next survey is undertaken. Both of these situations could affect the calculated HR. As a result, the real risks might be larger or smaller than the results reported in this paper. Moreover, this assumption has the potential to introduce misclassification bias into the study. Individuals might not use bed net consistently across time, although some studies have reported that bed net users tend to use the nets for a relatively long period [40], [41]. In addition, the study area is surrounded by Lake Victoria, which makes mosquitos available even in the dry season, as lagoons that are separated from the lake by sand bars become breeding places for mosquitos [42]. Based on these facts, we assumed that most habitants continuously used a bed net once they started using it. Furthermore, we used ACCM rates instead of malaria death rates to calculate the risks. This is due to the presence of ambiguous diagnoses for causes of death and the fact that ACCM is the best index to calculate the risk in a rural African location [43]. In terms of significance of the statistical tests used in this study, only linear trends were significant in our analyses. Generally, a trend test is likely to be more sensitive compared with a pairwise comparison to a reference group [27]. However, it may be helpful to report the estimate from a continuous analysis because it can detect a trend more powerful and insightful than a pairwise test across multiple groups [44]. To confirm our results, we need to have more observations in terms of the relation between child mortality and LLIN and young population densities. Furthermore, similar evaluation should be done in geographically different areas, because of geographical differences caused by environmental, cultural, economic and other related factors.

In conclusion, we observed that mortality risks between bed net users and non-bed net users were not significantly different. We found increasing linear trends for mortality risks among non-bed net users across LLIN density quartiles around each child as well as decreasing trends in risk across quartiles of young population densities around each child. When we consider worldwide bed net distribution and the fact that bed nets are sometimes used improperly, even when they are excessively distributed, it appears that a need exists for a system to monitor the current malaria situation globally in order to optimize malaria control programs with limited resources.

## Supporting Information

Table S1 Quartile values for LLIN and young population densities from a 100-meter radius model to a 3000-meter radius model.
(PDF)

Table S2 List of assets and factor scores used for the analysis.
(PDF)
