# Supplementary material for: Are Long-Lasting Insecticidal Nets Effective for Preventing Childhood Deaths among Non-Net Users? A Community-Based Cohort Study in Western Kenya
Source: PLoS One. 2012 Nov 19;7(11):e49604. doi: 10.1371/journal.pone.0049604 (PMC3501471; doi:10.1371/journal.pone.0049604)
Supplement: Table S1 — Quartile values for LLIN and young population densities from a 100-meter radius model to a 3000-meter radius model. (PDF) [file pone.0049604.s001.pdf]

Table S1. Quartile values for LLIN and young population densities from a 100-meter radius model to a 3000-meter radius model

1) LLIN densities in a circle around a child; number of LLIN per square kilometer (km^2)

Data set	Percentile	Radius from a child (meters)																													
		100	200	300	400	500	600	700	800	900	1000	1100	1200	1300	1400	1500	1600	1700	1800	1900	2000	2100	2200	2300	2400	2500	2600	2700	2800	2900	3000
For children sleeping without a bed net (A) in Figure 3	25%	0.0	15.9	14.1	11.9	11.5	11.5	10.4	10.4	10.2	9.9	9.7	9.3	9.0	8.4	8.5	8.3	8.3	8.2	7.9	7.7	7.6	7.6	7.5	7.3	7.1	6.9	6.7	6.6	6.4	6.3
	50%	63.7	39.8	31.8	29.8	28.0	26.5	25.3	22.9	21.6	20.4	19.2	18.1	17.3	16.6	16.0	15.4	15.3	15.0	14.9	14.5	14.2	13.9	13.8	13.6	13.3	13.1	13.1	13.1	13.1	13.2
	75%	159.2	103.5	77.8	67.6	57.3	50.4	46.8	43.8	41.3	39.5	38.7	37.8	36.4	35.1	33.4	32.2	30.7	30.2	30.2	29.5	29.2	28.6	27.9	27.5	27.7	27.2	26.6	25.9	25.6	25.4
For children sleeping without a bed net who lived far from health facilities (B) in Figure 3	25%	0.0	8.0	7.1	8.0	7.6	7.1	7.1	7.0	7.1	6.7	6.6	6.6	6.6	6.5	6.2	6.2	6.2	6.1	6.2	6.2	6.3	6.0	6.1	6.1	6.1	6.0	5.9	5.8	5.6	5.4
	50%	31.8	23.9	21.2	19.9	17.8	16.8	16.2	15.4	14.5	14.0	13.4	12.8	12.4	12.2	11.9	11.6	11.3	11.1	10.9	10.7	10.5	10.2	10.1	9.9	9.8	9.9	9.9	9.9	9.8	9.8
	75%	95.5	55.7	42.4	37.8	33.1	30.9	28.6	26.4	24.8	23.6	22.4	21.2	20.5	20.0	19.4	18.8	18.4	17.9	17.6	17.3	16.9	16.7	16.4	16.2	15.9	15.8	15.7	15.6	15.4	15.2
For trand analysis among children sleeping without a bed net who lived far from health facilities (A) in Figure 4	25%	0.0	8.0	7.1	8.0	7.6	7.1	7.1	7.0	7.1	6.7	6.6	6.6	6.6	6.5	6.2	6.2	6.2	6.1	6.2	6.2	6.3	6.0	6.1	6.1	6.1	6.0	5.9	5.8	5.6	5.4
	50%	31.8	23.9	21.2	19.9	17.8	16.8	16.2	15.4	14.5	14.0	13.4	12.8	12.4	12.2	11.9	11.6	11.3	11.1	10.9	10.7	10.5	10.2	10.1	9.9	9.8	9.9	9.9	9.9	9.8	9.8
	75%	95.5	55.7	42.4	37.8	33.1	30.9	28.6	26.4	24.8	23.6	22.4	21.2	20.5	20.0	19.4	18.8	18.4	17.9	17.6	17.3	16.9	16.7	16.4	16.2	15.9	15.8	15.7	15.6	15.4	15.2

* Gray collored cells are values in the best fit model among the 30 Cox PH models from 100-meter to 3000-meter radius models.

2) Population densities of young people between five years old and 20 years old in a circle around a child; number of young poeple per square kilometer (km^2)

Data set	Percentile	Radius from a child (meters)																													
		100	200	300	400	500	600	700	800	900	1000	1100	1200	1300	1400	1500	1600	1700	1800	1900	2000	2100	2200	2300	2400	2500	2600	2700	2800	2900	3000
For children sleeping without a bed net (A) in Figure 5	25%	191.0	143.2	123.8	115.4	108.2	102.6	98.1	92.5	88.0	83.4	78.9	74.5	71.8	68.9	66.5	64.5	63.1	61.7	60.0	58.6	57.4	56.0	54.6	53.5	52.5	51.3	50.0	49.1	48.2	47.5
	50%	350.1	246.7	208.7	181.0	163.0	149.4	137.1	126.8	118.7	113.0	107.3	103.2	98.9	94.8	91.0	88.2	85.6	83.1	80.7	78.1	75.9	73.9	71.8	70.3	68.9	67.4	65.9	64.6	63.6	62.5
	75%	732.1	461.5	367.8	312.3	269.9	246.7	228.7	207.4	189.4	179.5	168.9	161.8	155.4	149.9	145.1	139.1	134.2	129.9	124.5	119.3	116.8	113.4	111.7	109.5	107.4	104.5	102.0	99.9	98.8	98.4
For children sleeping without a bed net who lived far from health facilities (B) in Figure 5	25%	159.2	119.4	102.6	95.5	90.4	87.5	84.4	80.1	76.2	72.3	69.4	66.1	62.7	60.6	58.0	56.2	55.0	53.5	52.3	51.2	50.2	49.3	49.0	48.3	47.4	46.1	45.2	44.1	42.9	41.8
	50%	286.5	191.0	162.7	143.2	129.9	121.1	113.0	106.9	101.0	95.8	91.3	86.7	83.4	80.7	78.1	75.6	73.1	71.5	69.4	67.2	65.3	63.3	61.9	60.7	59.8	58.8	57.9	57.3	56.6	55.7
	75%	509.3	294.4	244.0	206.9	180.8	164.5	148.8	136.8	126.5	119.7	114.2	109.0	104.2	100.4	97.2	95.4	93.0	91.3	88.6	85.4	82.6	80.4	78.6	76.6	74.6	73.2	71.7	70.7	69.8	69.0
For tend analysis among children sleeping without a bed net who lived far from health facilities (A) in Figure 4	25%	159.2	119.4	102.6	95.5	90.4	87.5	84.4	80.1	76.2	72.3	69.4	66.1	62.7	60.6	58.0	56.2	55.0	53.5	52.3	51.2	50.2	49.3	49.0	48.3	47.4	46.1	45.2	44.1	42.9	41.8
	50%	286.5	191.0	162.7	143.2	129.9	121.1	113.0	106.9	101.0	95.8	91.3	86.7	83.4	80.7	78.1	75.6	73.1	71.5	69.4	67.2	65.3	63.3	61.9	60.7	59.8	58.8	57.9	57.3	56.6	55.7
	75%	509.3	294.4	244.0	206.9	180.8	164.5	148.8	136.8	126.5	119.7	114.2	109.0	104.2	100.4	97.2	95.4	93.0	91.3	88.6	85.4	82.6	80.4	78.6	76.6	74.6	73.2	71.7	70.7	69.8	69.0

* Gray collored cells are values in the best fit model among the 30 Cox PH models from 100-meter to 3000-meter radius models.
