# Supplementary material for: Are Long-Lasting Insecticidal Nets Effective for Preventing Childhood Deaths among Non-Net Users? A Community-Based Cohort Study in Western Kenya
Source: PLoS One. 2012 Nov 19;7(11):e49604. doi: 10.1371/journal.pone.0049604 (PMC3501471; doi:10.1371/journal.pone.0049604)
Supplement: Table S2 — List of assets and factor scores used for the analysis. (PDF) [file pone.0049604.s002.pdf]

Table S2. List of assets and factor scores used for the analysis

Asset Variable	asset factor score	Mean	Std. Dev.	has asset	Does not have asset
Has car	0.048	0.003	0.056	0.860	-0.003
Has fridgerator	0.063	0.002	0.046	1.354	-0.003
Has television	0.111	0.073	0.260	0.397	-0.031
Has stereo set	0.038	0.017	0.128	0.290	-0.005
Has ironbox	0.044	0.220	0.415	0.083	-0.024
Has cellular phone	0.150	0.400	0.490	0.184	-0.123
Has motorcycle	0.075	0.020	0.140	0.527	-0.011
Has fan	0.028	0.007	0.083	0.331	-0.002
Has radio	0.073	0.771	0.420	0.040	-0.133
Has bicycle	-0.023	0.282	0.450	-0.036	0.014
Has sofa	0.024	0.672	0.469	0.017	-0.035
Has table	-0.037	0.937	0.242	-0.010	0.145
Has electricity	0.125	0.015	0.120	1.024	-0.015
Has bed	-0.037	0.945	0.228	-0.009	0.152
Has livestock	-0.174	0.387	0.487	-0.219	0.138
Has poultry	-0.228	0.635	0.481	-0.173	0.301
Has sewing machine	0.016	0.015	0.123	0.126	-0.002
Has gas	0.197	0.047	0.211	0.888	-0.044
If has rudimentary walls	-0.300	0.609	0.488	-0.241	0.375
If has earth as principal floor in dwelling	-0.320	0.673	0.469	-0.223	0.459
If has natural material roofing	-0.129	0.118	0.323	-0.352	0.047
If uses a lantern for lighting	0.133	0.345	0.475	0.184	-0.097
If uses electricity for lighting	0.144	0.015	0.122	1.157	-0.018
If uses tin lamp for lighting	-0.169	0.604	0.489	-0.137	0.208
If uses other for lighting	0.006	0.029	0.168	0.036	-0.001
If uses charcoal for cooking heat	0.358	0.190	0.392	0.739	-0.174
If uses firewood for cooking heat	-0.364	0.783	0.412	-0.191	0.691
If uses other for cooking heat	0.049	0.020	0.140	0.341	-0.007
If household own land	-0.346	0.718	0.450	-0.217	0.552
If household lease land	0.334	0.219	0.413	0.632	-0.177
If household land is other category	0.068	0.057	0.231	0.278	-0.017
Survey period I	0.018	0.331	0.471	0.025	-0.012
Survey period II	0.001	0.225	0.417	0.001	0.000
Survey period III	-0.007	0.222	0.415	-0.012	0.003
Survey period IV	-0.014	0.222	0.416	-0.027	0.008
If toilet is flush one	0.012	0.000	0.015	0.840	0.000
If toilet is pit latrine	0.152	0.138	0.345	0.380	-0.061
If toilet is ventilated pit latrine	0.064	0.004	0.064	1.001	-0.004
